# Optimum timing of emergency cholecystectomy for acute cholecystitis in England: population-based cohort study

**DOI:** 10.1007/s00464-018-6537-x

**Published:** 2019-04-04

**Authors:** Tom Wiggins, Sheraz R. Markar, Hugh MacKenzie, Omar Faiz, Dipankar Mukherjee, David E. Khoo, Sanjay Purkayastha, Ian Beckingham, George B. Hanna

**Affiliations:** 1 Department Surgery and Cancer, Imperial College London, London, UK; 2 Barking Havering and Redbridge NHS Trust, Queen’s Hospital, London, UK; 3 St Mark’s Hospital and Academic Institute, Harrow, UK; 4 Queen’s Medical Centre, Nottingham University Hospitals NHS Trust, Nottingham, UK; 5 Division of Surgery, Department of Surgery and Cancer, Imperial College London, St Mary’s Hospital, 10th Floor QEQM Building, South Wharf Road, London, W2 1NY UK

**Keywords:** Cholecystitis (MeSH), Cholecystitis, acute (MeSH), Emergency cholecystectomy

## Abstract

**Background:**

Cholecystectomy on index admission for acute cholecystitis is associated with improved patient outcomes. The timing of intervention is mainly driven by service provision. This population-based cohort study aimed to evaluate timing of emergency cholecystectomy in England.

**Methods:**

Data from all consecutive patients undergoing surgery for acute cholecystitis on index admission in England from 1997 to 2012 were captured from the Hospital Episodes Statistics database. Data were analysed based on whether patients underwent surgery 0–3 days, 4–7 days or ≥ 8 days from admission. Outcome measures were rate of post-operative biliary complications, conversion to open and length of stay.

**Results:**

Forty-three thousand eight hundred and seventy patients underwent emergency cholecystectomy. 64.6% of patients underwent surgery between days 0 and 3 of admission, 24.3% between days 4–7 and 11.0% had surgery after day 8. Patients undergoing early surgery had significantly reduced rates of intra-operative laparoscopic conversion to open (0–3 days: 3.6%; 4–7 days: 4.0%; ≥ 8 days 4.7%, *p* = 0.001), post-operative ERCP (0–3 days: 1.1%; 4–7 days: 1.5%; ≥ 8 days 1.9%, *p* < 0.001) and bile duct injury (0–3 days: 0.6%; 4–7 days: 1.0%; ≥ 8 days 1.8%, *p* < 0.001). Early cholecystectomy was also associated with a shorter post-operative length of stay (LOS) [0–3 days group: median post-operative LOS 3 days (IQR: 1–6); 4–7 days group: 3 days (IQR 2–6); ≥ 8 days group: 4 days (IQR 2–9) (*p* < 0.001)]. High-volume centres undertook a significantly greater proportion of cholecystectomies within 3 days of presentation (high-volume: 67.3%; medium-volume: 64.8%; low-volume: 61.2%). In multivariate analysis greater time to surgery was independently associated with increased risk of post-operative ERCP and bile duct injury.

**Conclusions:**

Early cholecystectomy within 3 days of admission reduces intra-operative conversion, post-operative biliary complications and length of stay. Centres undertaking the greatest numbers of emergency cholecystectomies perform a larger proportion within 3 days of admission.

Acute cholecystitis is a common complication of gallbladder stones. Cholecystectomy represents the only definitive management strategy for symptomatic gallstone disease. Undertaking cholecystectomy during index admission for acute cholecystitis is associated with reduced long-term biliary complications, shorter total admission length of stay and lower overall treatment costs [1–7].

Guidelines from the World Society of Emergency Surgery (WSES) recommend that early laparoscopic cholecystectomy should be performed as soon as safely possible but can be performed up to 10 days from symptom onset [8]. In the United Kingdom the National Institute for Health and Care Excellence (NICE) recommend that patients with acute cholecystitis should receive cholecystectomy within 7 days of diagnosis [9]. The utilization of emergency cholecystectomy has not been universally adopted in the United Kingdom with only 15.7% of patients with acute cholecystitis undergoing cholecystectomy on index admission (compared to 52.7% in the United States) [10], and there is a wide variation in the utilization of emergency cholecystectomy across hospitals in the United Kingdom [11].

Most current studies, although limited by sample size, advocate an early approach to emergency cholecystectomy with previous evidence suggesting that undertaking surgery within 72 h of symptom onset or hospital admission reduces operative time, decreases length of hospital stay, is associated with fewer adverse post-operative outcomes and reduced mortality compared to patients undergoing surgery later in their clinical course [12–14]. However, other studies have demonstrated that emergency cholecystectomy can be undertaken up to 7 days from symptom onset with equivalent outcomes [15].

This national population-based cohort study aimed to evaluate how timing of emergency cholecystectomy affects outcomes for patients in England. The effect of hospital volume of emergency cholecystectomy upon timing of surgery and outcomes has also been examined. The primary end point was post-operative biliary complications while secondary end points were conversion to open and length of hospital stay.

## Materials and methods

Data were derived from the Hospital Episode Statistics (HES) database [16]. This is an administrative dataset that collects patient-level data from all National Health Service (NHS) hospitals in England. It captures all patients treated in public sector hospitals and a minority of patients treated in privately funded institutions. Patients are linked to a unique HES identifier, which allows all of their hospital admissions to be tracked throughout the dataset. Permissions for the comparison of anonymized administrative data were obtained from the National Information Governance Board for Health and Social Care in England.

### Coding of data

Relevant International Classification of Disease (ICD-10) codes were used to identify all patients who were admitted as an emergency for the treatment of acute cholecystitis (ICD-10 Codes K80.0, K80.1, K80.4, K81.0, K81.8, K81.9), between 1st January 1997 and 31st December 2012. Only patients identified as emergency admissions were included in this analysis using the admission codes (method of admission 21–28). Patients primarily admitted with other gallstone related disorders, including biliary colic, acute cholangitis and acute pancreatitis were excluded from this study. Local verification of ICD-10 codes used for diagnosis and patient allocation was performed as part of the quality assurance for the data included.

### Allocation to treatment groups

Those patients who underwent cholecystectomy during index admission were identified using the Office of Population Censuses and Surveys Classification of Surgical Operations and Procedures 4th revision (OPCS-4) codes (supplementary material). Patients were allocated to three treatment groups based upon the elapsed time between admission and cholecystectomy. Definition of treatment groups was undertaken prior to data analysis through consensus between authors to identify clinically relevant threshold values. Treatment groups were defined as:

1. Patients undergoing cholecystectomy from day of admission to day three following admission (0–3 days)
2. Patients receiving cholecystectomy between days four and seven following admission (4–7 days)
3. Patients undergoing cholecystectomy greater than 8 days following admission (≥ 8 days)

All patients requiring pre-operative primary intervention with endoscopic retrograde cholangio-pancreatogram (ERCP) were excluded from analysis due to the potential influence of need for ERCP affecting time delay to cholecystectomy.

Post-operative biliary complications were defined as need for post-operative ERCP (as treatment of post-operative bile leak or retained stones) or bile duct injury (defined by need for biliary reconstruction). These interventions were identified through the relevant OPCS-4 codes (supplementary material).

### Exposure

The exposure under investigation was timing of cholecystectomy on index admission for acute cholecystitis: either 0–3 days following admission, 4–7 days or ≥ 8 days.

### Outcomes

Primary outcome measures were the rate of post-operative biliary complications following emergency cholecystectomy [including need for post-operative ERCP or bile duct injury (defined by need for bile duct reconstruction)]. The secondary outcome measures were intra-operative conversion to open surgery and post-operative length of stay (LOS) following emergency cholecystectomy.

Outcomes were also evaluated based upon hospital volume of emergency cholecystectomy. Hospital volume thresholds were established through tertile analysis of the current dataset to divide hospitals into low-volume (1–171 cases over study-period), medium-volume (172–316 cases over study-period) and high-volume (≥ 317 cases over study-period) for emergency cholecystectomy.

### Statistical analysis

Statistical analysis was performed using SPSS version 23.0 software [Statistical Package for the Social Sciences software, Version 23, SPSS Chicago (IL), USA]. Univariate comparisons between treatment groups were made with Chi-squared test for discrete variables and Kruksal-Wallis test for continuous variables. Multivariable logistic regression analyses were performed to evaluate the positive or negative association of cholecystectomy timing with post-operative complications (need for post-operative ERCP or bile duct injury). Confounding factors adjusted for in this analysis included: age (under 70 years, or over 70 years), sex (male or female), Charlson comorbidity index score (< 2 or ≥ 2) and hospital volume [1–171 cases (low-volume), 172–316 cases (medium-volume) and ≥ 317 cases (high-volume)].

## Results

### Population characteristics

Over the 16-year period from 1997 to 2012 there were 43,870 patients in England who underwent emergency cholecystectomy on index admission. Table 1 demonstrates the patient demographic details for each of the treatment groups. Patients undergoing cholecystectomy at a time-point more than eight days following admission were significantly older [Median age in ≥ 8 days group 61, compared to 54 in both 0–3 days and 4–7 days groups (*p* < 0.001)] and had significantly more co-morbidities [Charlson comorbidity index ≥ 2 in 4.8% in 0–3 days group, 4.5% in 4–7 days group and 8.5% of ≥ 8 days group (*p* < 0.001)] (Table 1).

**Table 1:** Patient demographics, cholecystectomy timing by hospital volume, post-operative complications and post-operative length of stay (LOS)

Timing of cholecystectomy	0–3 days (%)	4–7 days (%)	≥ 8 days (%)	*p* value
Patient number	28,356 (64.6)	10,673 (24.3)	4841 (11.0)	
Age [median (IQR)]	54 (39–68)	54 (39–68)	61 (45–73)	< 0.001
Age ≥ 70	6346 (22.4)	2337 (21.9)	1571 (32.5)	< 0.001
Sex				
Male	9136 (32.2)	3132 (29.3)	1565 (32.3)	< 0.001
Female	19,220 (67.8)	7541 (70.7)	3276 (67.7)	
CCI				
< 2	26,984 (95.2)	10,188 (95.5)	4429 (91.5)	< 0.001
≥ 2	1372 (4.8)	485 (4.5)	412 (8.5)	
Hospital volume*				
0–171	8676 (61.2)	3480 (24.7)	1922 (13.7)	< 0.001
172–316	9597 (64.8)	3605 (24.4)	1601 (10.8)	
≥ 317	10,083 (67.3)	3588 (23.8)	1318 (8.8)	
Laparoscopic	18,558 (65.4)	6994 (65.5)	2760 (57.0)	< 0.001
Conversion to open	1021 (3.6)	427 (4.0)	228 (4.7)	0.001
Post-operative ERCP	318 (1.1)	162 (1.5)	94 (1.9)	< 0.001
Post-operative CBD reconstruction	159 (0.6)	106 (1.0)	89 (1.8)	< 0.001
Post-operative LOS (median (IQR))	3 (1–6)	3 (2–6)	4 (2–9)	< 0.001

*Calculated by hospital volume group

### Timing of cholecystectomy

Overall the majority of patients underwent cholecystectomy within 3 days of admission (*n* = 28,356, 64.6%). 10,673 patients underwent cholecystectomy between 4 and 7 days following admission (24.3%) and 4841 received surgery at a time interval of greater than 8 days (11.0%) (Table 1).

### Primary outcomes

**Fig. 1:**
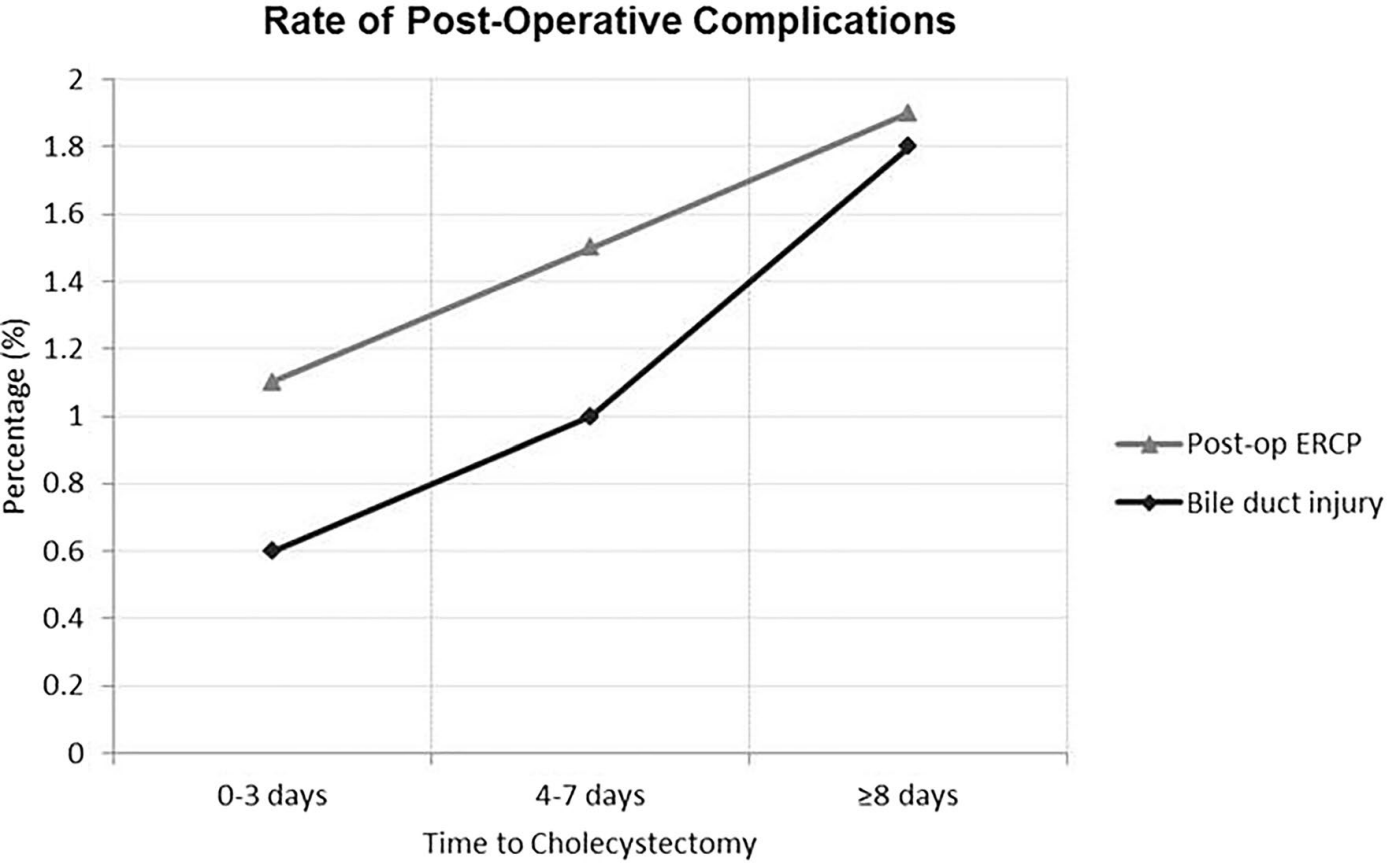
Post-operative biliary complications including need for post-operative ERCP and bile duct injury, stratified by time from admission to cholecystectomy (0–3 days, 4–7 days and ≥ 8 days)

There were significantly fewer post-operative biliary complications in patients undergoing cholecystectomy within 3 days of admission. Post-operative ERCP was required in 1.1% of this group of patients, compared to 1.5% in the 4–7 days and 1.9% in the ≥ 8 days groups (*p* > 0.001). There was also a significantly reduced rate of bile duct injury requiring common bile duct reconstruction in the 0–3 days group (0.6%) compared to the 4–7 days (1.0%) and ≥ 8 days (1.8%) groups (*p* < 0.001) (Table 1; Fig. 1). Over the study-period there was a significant reduction in the requirement for post-operative ERCP [1.4% (1997–2000) to 1.0% (2009–2012) (*p* < 0.001)] and post-operative common bile duct reconstruction [1.7% (1997–2000) to 0.3% (2009–2012) (*p* < 0.001).

### Secondary outcomes

The rate of a primary laparoscopic approach to cholecystectomy was increased in the early cholecystectomy groups (0–3 days: 65.4%; 4–7 days: 65.5%) compared with those receiving surgery at ≥ 8 days (57.0%; *p* < 0.001). The rate of intra-operative conversion was reduced in the early cholecystectomy group [0–3 days: 3.6%; 4–7 days: 4.0%; ≥ 8 days: 4.7% (p < 0.001)]. The overall prevalence of intra-operative conversion did not change during the study-period [4.0% (1997–2000) to 4.2% (2009–2012) *p* = 0.064). Early cholecystectomy within 3 days of admission was also associated with a shorter post-operative length of stay (LOS) [median post-operative LOS 3 days (IQR: 1–6) compared to the patients who underwent cholecystectomy later during admission [4–7 days group median post-operative LOS 3 days (IQR 2–6); ≥ 8 days group median post-operative LOS 4 days (IQR 2–9) (*p* < 0.001)] (Table 1).

### Hospital volume

**Fig. 2:**
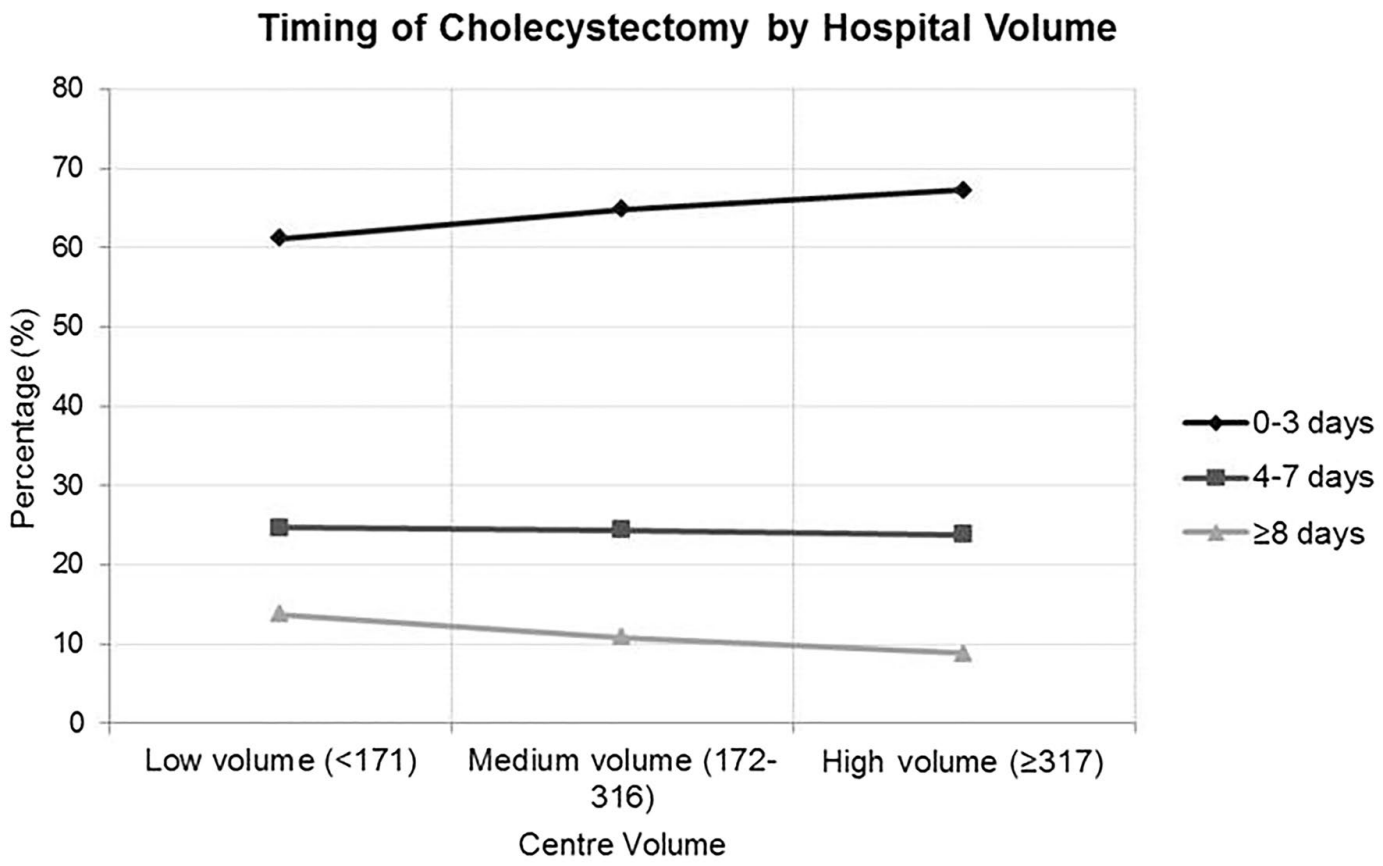
Timing of cholecystectomy stratified by hospital volume (low-volume < 171 procedures; medium-volume 172–316 procedures; high-volume ≥ 317 procedures)

Hospitals were divided into three volume groups based upon tertile analysis of number of emergency cholecystectomies performed during the study-period. High-volume hospitals were those who performed greater than 317 emergency cholecystectomies during the study-period. Medium-volume units performed between 172 and 316 emergency cholecystectomies during this period and those considered low-volume undertook less than 171 procedures. Higher volume hospitals performed significantly more cholecystectomies within 3 days of admission [67.3% compared to 64.8% in medium-volume and 61.2% in low-volume units (*p* < 0.001)]. These high-volume centres also performed significantly less procedures at a time-point greater than 8 days following admission [high-volume 8.8%; medium-volume 10.8%; low-volume 13.7% (*p* < 0.001)] (Table 1; Fig. 2).

### Multivariate analysis

Multivariate analysis was utilized to establish factors associated with need for post-operative ERCP. Charlson comorbidity index above 2 was significantly associated with decreased rate of post-operative ERCP (Hazard ratio (HR) 0.51 [95% confidence interval (CI) 0.32–0.84) (*p* = 0.008)]. Increasing time to cholecystectomy was independently associated with increased need for post-operative ERCP. With the 0–3 days group considered as reference, those undergoing cholecystectomy at [4–7 days had a HR of 1.36 (95% CI 1.12–1.64 (*p* = 0.002) and ≥ 8 days HR 1.74 (95% CI 1.38–2.20 (*p* < 0.001)] (Table 2). Increasing hospital volume was associated with a significant decrease in the post-operative ERCP rate, with low-volume (1–171 cases) as reference, high-volume centres (≥ 317 cases) were associated with a significantly reduced risk of post-operative ERCP (HR 0.81, 95%CI 0.65–0.95) (Table 2).

**Table 2:** Multivariate analysis to evaluate factors influencing need for post-operative ERCP

Factor	Hazard ration (95% confidence interval)	*p* value
Age ≥ 70		
No (ref)	–	–
Yes	1.08 (0.89–1.31)	0.447
Sex		
Male (ref)	–	–
Female	0.94 (0.78–1.12)	0.487
CCI		
< 2 (ref)	–	–
≥ 2	0.51 (0.32–0.84)	0.008
Hospital volume		
1–171 (ref)	–	–
172–316	0.94 (0.86–1.57)	0.234
≥ 317	0.81 (0.65–0.95)	0.045
Pre-operative duration		
0–3 days (ref)	–	–
4–7 days	1.36 (1.12–1.64)	0.002
≥ 8 days	1.74 (1.38–2.20)	< 0.001

During multivariate analysis the risk of bile duct injury requiring common bile duct reconstruction was increased with age ≥ 70 years [(HR 2.13, 95% CI 1.71–2.65; *p* < 0.001)], and Charlson comorbidity index above 2 [(HR 3.37 (95% CI 2.57–4.43; *p* < 0.001)). Female patients had a significantly reduced risk of this complication [(HR 0.73 (95% CI 0.59–0.90; *p* = 0.004)]. Increased time to cholecystectomy was also independently associated with increased risk of bile duct injury. Utilizing the 0–3 days group as reference those undergoing cholecystectomy at 4–7 days following admission [(HR 1.82, 95% CI 1.42–2.34; *p* < 0.001)] or ≥ 8 days following admission [HR 2.76 (95% CI 2.12–3.60; *p* < 0.001)] had significantly increased risk of bile duct injury. Increasing hospital volume was associated with a significant decrease in the common bile duct reconstruction rate (low-volume as reference; medium-volume HR 0.68, 95% CI 0.53–0.87; high-volume: HR 0.64, 95% CI 0.50–0.83) (Table 3).

**Table 3:** Multivariate analysis to evaluate factors influencing need for bile duct injury

Factor	Hazard ration (95% confidence interval)	*p* value
Age ≥ 70		
No (ref)	–	–
Yes	2.13 (1.71–2.65)	< 0.001
Sex		
Male (ref)	–	–
Female	0.73 (0.59–0.90)	0.004
CCI		
< 2 (ref)	–	–
≥ 2	3.37 (2.57–4.43)	< 0.001
Hospital volume		
1–171 (ref)	–	–
172–316	0.68 (0.53–0.87)	0.002
≥ 317	0.64 (0.50–0.83)	0.001
Pre-operative duration		
0–3 days (ref)	–	–
4–7 days	1.82 (1.42–2.34)	< 0.001
≥ 8 days	2.76 (2.12–3.60)	< 0.001

## Discussion

This national population-based cohort study including data from 43,870 patients has demonstrated that early intervention by undertaking emergency cholecystectomy within 3 days of admission is associated with fewer post-operative biliary complications, reduced need for intra-operative conversion and shorter post-operative length of stay. Patient’s undergoing emergency cholecystectomy more than 8 days following presentation were associated with the highest rates of post-operative ERCP and bile duct injury, as well as increased post-operative length of stay. These findings are in keeping with previous studies, which have identified that early intervention for emergency cholecystectomy is generally associated with improved patient outcomes. A French national healthcare database study identified that patient’s undergoing cholecystectomy on days 1–3 of admission had improved post-operative outcomes (reduced intensive care admissions, re-operation or post-operative sepsis) when compared to those undergoing surgery from day 5 onwards [13]. Additional studies have advocated that patients with cholecystitis should undergo surgery as soon as safely feasible, and that immediate surgery on the day of admission is associated with improved outcomes [6, 7, 17]. Undertaking cholecystectomy within the first 24–48 h of hospital admission is also associated with reduced hospital costs [7, 14].

In the current study, increasing centre volume was independently associated in multivariate analysis with reduced risk of need for post-operative ERCP or bile duct injury. This is consistent with the findings of previous studies which demonstrated that units or individual surgeons who undertake large volumes of cholecystectomies are associated with improved outcomes relating to decreased length of stay and fewer conversion to open procedures [18, 19], as well as reduced re-operation rates and readmissions [18]. In addition to reduced incidence of complications, hospitals that undertook the largest volume of emergency cholecystectomies in the current study (> 317 cases, ‘high-volume’) undertook a larger proportion of emergency cholecystectomies within 3 days of presentation (67.3%) compared to other centres (rate of cholecystectomy within 3 days for medium-volume centres 64.8%; low-volume centres 61.2% (*p* < 0.001). This suggests that hospitals undertaking a larger volume of emergency cholecystectomies appear to have greater provision to undertake cholecystectomy within 3 days of admission. This could relate to greater infrastructure in these units including access to radiology services for diagnosis, theatre availability and presence of appropriately experienced surgeons to undertake emergency cholecystectomy. A previous population-based study in the United Kingdom and Ireland identified that there were a number of hospital related factors which could influence whether a unit may undertake emergency cholecystectomy [11]. Emergency cholecystectomies were more likely to be performed in university hospitals (*p* < 0.001), specialist hepatobiliary units (*p* < 0.001), hospitals with more beds (*p* < 0.001) and hospitals that performed a greater total number of cholecystectomies (*p* < 0.001). Specialist oesophagogastric or hepatobiliary surgeons were also more likely to perform a greater proportion of cholecystectomies as emergency procedures than as delayed operations (*p* = 0.011) [11]. Although these factors may have influenced the likelihood of patients receiving emergency cholecystectomy the same study identified that their modelling accounted for only 65 per cent of the variation in the provision of emergency cholecystectomy identified [11]. This indicates there are other potentially complex factors which influence service delivery for emergency cholecystectomy. The Royal College of Surgeons of England is currently completing a large-scale national quality improvement project through the Cholecystectomy Quality Improvement Collaborative (Chole-QuIC) [20], which aims to develop a translatable model for delivery of emergency cholecystectomy services. This project may help to reduce the variability in the provision of emergency cholecystectomy across the United Kingdom and help identify the specific factors, which can expedite the development of these services.

In the current study there was a 27% increased relative risk of bile duct injury in male patients undergoing emergency cholecystectomy. Previous studies have identified that male gender appears to be an independent risk factor for adverse outcomes following surgery for acute cholecystitis [21, 22]. Male patients undergoing emergency cholecystectomy have been demonstrated to have greater risk of mortality, increased complications, longer hospital stay and higher overall costs compared to females [21]. These gender differences appear to be multifactorial and it has been hypothesized that there may be contributions from men having a delay in time to diagnosis, be older at the time of surgery and have increased risk of conversion to open surgery [21, 23, 24].

Patient’s undergoing emergency cholecystectomy within 3 days of admission had a significantly lower risk of requiring conversion to open surgery [Conversion rate 0–3 days: 3.6%; 4–7 days: 4.0%; ≥ 8 days: 4.7% (*p* < 0.001)]. This finding is consistent with previous studies demonstrating that delaying surgery for acute cholecystitis can increase need for open conversion, and those patients waiting more than 6 days for surgery have a three-fold increased risk of conversion to an open procedure compared to those undergoing emergency cholecystectomy on the day of admission [6]. It is likely that this increased risk of open surgery is due to increased operative difficulty in delayed cases. Open cholecystectomy for acute cholecystitis is associated with increased post-operative morbidity (particularly increased wound infection and pneumonia rates), higher mortality rates and prolonged length of stay [25]. By increasing the proportion of patients undergoing early surgery for acute cholecystitis within 3 days of admission it may be possible to reduce the requirement of conversion to open cholecystectomy.

The population-based design with virtually complete inclusion of all eligible patients undergoing emergency cholecystectomy in England is a significant strength of this study. The large sample size enables assessment of overall outcomes at a national level, and provides an overall evaluation of timing of emergency cholecystectomy between hospitals that undertake this procedure regularly (and may have appropriate infrastructure in place to facilitate early surgical intervention) and those hospitals which perform emergency cholecystectomy less frequently. However, as a large national database study the results generated are dependent upon the reliability of the methodology and accuracy of data collection, which is a limitation shared by all national administrative datasets. In the current study time to cholecystectomy was measured from the point of patient presentation to hospital, rather than symptom onset. Patients may have been experiencing symptoms for a variable length of time prior to hospital admission and this factor may have influenced operative difficulty and clinical outcomes. However, time of presentation has been successfully utilized as the initial time-point in previous national based studies investigating emergency cholecystectomy [6, 13, 14, 17]. Unfortunately in the current study, data were not available regarding operative time and this factor may have provided additional insight regarding operative difficulty, and whether procedures were more challenging with increasing time from admission to surgery. However, the current study demonstrated that increasing time to cholecystectomy was associated with increasing risk of need for conversion to open procedure and this would therefore indicate that surgery became more challenging in more delayed cases. Due to the absence of a separate ICD-10 code for Mirizzi syndrome it was not possibly to specifically identify these cases. A small proportion of the bile duct injury group may have represented Mirrizi syndrome patients who required bile duct repair due to the nature of their disease. Given the absence of a specific diagnostic code for Mirizzi syndrome within the current dataset it was not possible to exclude these cases from the bile duct injury group.

In conclusion this study has demonstrated that early cholecystectomy within 3 days of admission is associated with reductions in post-operative complications, intra-operative conversion and post-operative length of stay. Centres performing the greatest numbers of emergency cholecystectomies are able to perform a larger proportion within 3 days of initial admission. By improving patient pathways and available infrastructure to facilitate widespread adoption of emergency cholecystectomy in the United Kingdom it may be possible to further increase the proportion of patients receiving surgery within 3 days of admission and therefore improve clinical patient outcome.
